# Tree-Ring Dating of the Reshui-1 Tomb in Dulan County, Qinghai Province, North-West China

**DOI:** 10.1371/journal.pone.0133438

**Published:** 2015-08-05

**Authors:** Mingqi Li, Xuemei Shao, Zhi-Yong Yin, Xinguo Xu

**Affiliations:** 1 Key Laboratory of Land Surface Pattern and Simulation, Institute of Geographic Sciences and Natural Resources Research, Chinese Academy of Sciences, Beijing, China; 2 Chinese Academy of Sciences Center for Excellence in Tibetan Plateau Earth System Sciences, Beijing, China; 3 Department of Environmental and Ocean Sciences, University of San Diego, San Diego, California, United States of America; 4 Qinghai Provincial Institute of Cultural Relics and Archaeology, Xining, China; Chinese Academy of Sciences, CHINA

## Abstract

Tuyuhun and Tubo were two important states that thrived in north-western China during AD 311-900 in parallel with the Han Chinese dynasties of Sui and Tang periods. The Reshui Tomb Cluster located in Dulan County of the north-eastern Tibetan Plateau is an important cultural relic of the Tuyuhun-Tubo age. The official excavations of the Reshui tombs were regarded as top events in archaeology in the 1980s and 1990s in China. The Reshui-1 Tomb is the largest one among the tombs in the area. Since its excavation, there have been debates on whether the owner of the tomb belonged to the Tuyuhun or Tubo ethnicity. Therefore, accurately dating the Reshui-1 Tomb has a critical place in studying the Tubo and Tuyuhun histories. We collected 7 discs and 11 increment cores of Qilian juniper (*Juniperus przewalskii* Kom.) from the exposed and fallen beams of the roof of the Reshui-1Tomb. The lengths of the 16 tree-ring records are between 69 and 152 years. Based on a previously developed master dating chronology using Qilian juniper samples from the eastern Qaidam Basin, the calendar dates of the 16 specimens were determined by the COFECHA program and visual dating procedure. The average inter-series correlation among the dated sample series is 0.696, indicating good quality of cross-dating. The year of the outermost rings is AD 715 for the 7 discs and 4 out of the 9 increment cores. Moreover, the ring-width variations of the samples are consistent with the existing chronologies from the region. The presence of late-wood of AD 715 in the samples indicated that the Reshui-1 Tomb was completed in late AD 715 or early 716, which means that the Reshui-1 Tomb was finished in the Tubo age. This date provides direct evidence for archaeologists to determine the owner’s ethnicity and identify of the Reshui-1 Tomb.

## Introduction

**Fig 1:**
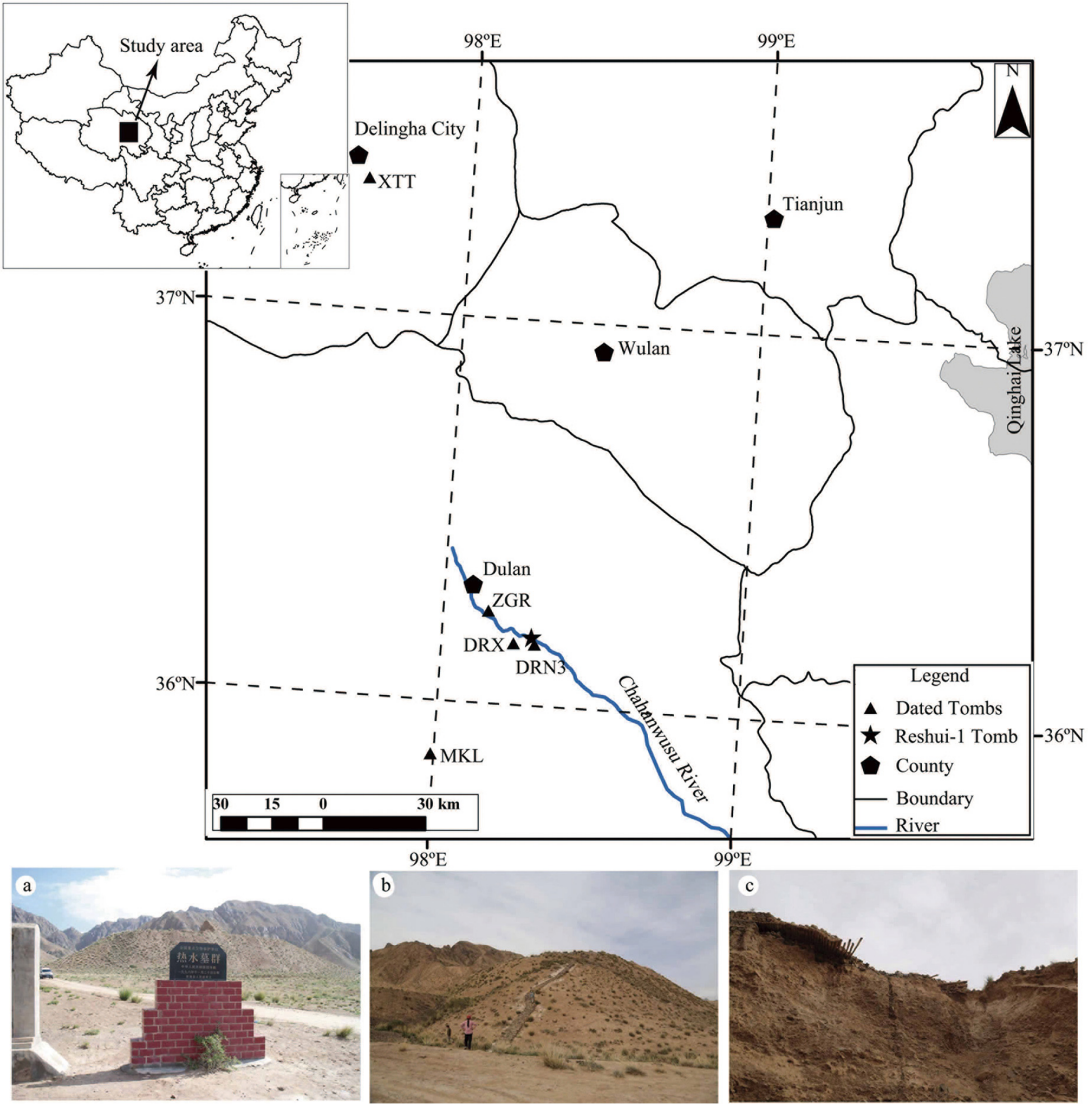
Location and Pictures of Reshui-1 tomb relative to the locations of the dated tombs (No. XTT, ZGR, DRX and DRN3) in Dulan and Delingha areas. XTT includes four tombs [26] and DRX includes 7 tombs (00DRXM3, 00DRXM8, 00DRXM10, 00DRXM14, 00DRXM19, 00DRXM21, 00DRXM23) [26]. Picture a) Signage of the Reshui Tomb Cluster; b) Full view of the Reshui-1 Tomb (from west); c) Collapsed portion of the tomb roof and the exposed roofing beams.

Since the early 1900s, there have been numerous rescue excavations of ancient tombs by governmental archaeologists in China. Even though China has a long documented history, these tombs still provided new information about the tomb owners and various aspects of culture and political and economic conditions for the specific time periods. In 1983, governmental archaeologists excavated the Reshui-1 (also known as Xuewei No.1) Tomb in Dulan County, Qinghai Province in the north-eastern Tibetan Plateau, North-west China (Fig 1), which was claimed as one of the “Top Six Archaeological Discoveries of 1983 in China” [1]. The Reshui Tomb Cluster was one of the most important cultural relics of the Tuyuhun age (AD 329–663) [2] and Tubo age (early 7^th^ century to the second half of the 9^th^ century) [3]. The official excavation of the Reshui Tomb Cluster was regarded as one of the “Top Ten Archaeological Discoveries of 1996 in China” [1]. The Reshui-1 Tomb lies in the center of the Reshui Tomb Cluster and has the largest dimension among all tombs in the area. In fact it is also the largest one among the tombs of the Tuyuhun-Tubo age in Qinghai Province [4], suggesting the owner of the tomb as a high-ranking official or an important aristocrat. Therefore, it has a very important place in studying the Tubo and Tuyuhun histories.

Tuyuhun was a nomadic tribe of approximately 1700 families that migrated from northeastern China to this region after AD 329 [5]. It reached its peak during the 5^th^ century, with influences strong enough to compete with the Han Chinese dynasties (Sui and Tang) in this region [5]. Around the turn of the 7^th^ century, however, the ancient Tibetan Kingdom, Tubo Kingdom, grew stronger and eventually conquered Tuyuhun in AD 663 and took over all the land of Tuyuhun [5]. Based on the tomb structure and unearthed artifacts, the age of the Reshui-1 Tomb was determined to be from the late 7^th^ century to mid- 8^th^ century [4, 6]. Nonetheless, an exact year could not be designated to the tomb at this moment. As of now, no radiocarbon dates have been published and even if they are available, they could not pinpoint the exact year of tomb construction. Without an exact date of the tomb construction, the tomb owner and his/her ethnicity cannot be determined, which leaves an important gap for the studies of the Tuyuhun and Tubo histories.

In the past decades, tree-rings analysis has been successfully employed to archaeological dating [7–13], including dating pre-historical relic sites [14–18], historical structures [19–26], tombs and burial sites [7, 27], artifacts [28–33], and past events (e.g., shipwrecks) [34].

Qilian juniper (*Juniperus przewalskii* Kom.), growing in the north-eastern part of the Qinghai-Tibetan Plateau, provides a unique opportunity to develop long tree-ring chronologies in China [35–38]. In the past decades, Qilian juniper has been employed to study the paleoclimate and construct dating chronologies, using samples from both living trees and archaeological wood unearthed from ancient tombs [39–46]. In the meantime, dendrochronology has been used to determine the ages of ancient tombs in the Dulan region. For example, using archaeological Qilian juniper samples, Wang [8] determined that 7 tombs (00DRXM3, 00DRXM8, 00DRXM10, 00DRXM14, 00DRXM19, 00DRXM21, 00DRXM23, Fig 1) of the Reshui Tomb Cluster were built between AD 611 and AD 784. Additionally, Wang et al. [7] presented tree ring dates for three additional tombs in Dulan, including a tomb (DRN3) on the south bank of Reshui (Reshuinanan) dated to AD 784, a tomb (MKL) near the village of Mokeli to AD 783, and a tomb (ZGR) in Zhigari to AD 789. Wang et al. [26] also presented ages for four tombs (XTT) in Xiatatu from AD 756 to AD 790 near Delingha, approximately 120 km north of Dulan (Fig 1). All these dates were based on dendrochronological work summarized in Shao et al. [46].

Although previous studies provided the context and general timeframe for the Reshui-1 Tomb, the exact date of construction of this most important archaeological site of the region is still missing at the time of the current study. As a result, there is not a firm conclusion on the owner’s ethnicity and identify. For example, based on historical documents, it has been speculated that the owner of the Reshui-1 Tomb may be a Tuyuhun Khan or a Tubo minister/governor who died from the late 7^th^ century to the mid-8^th^ century [4, 47, 48]. Therefore, the goal of this study is to determine the exact date for the Reshui-1 Tomb using Qilian juniper tree-ring data by cross-dating the samples from the tomb roof to an existing master dating chronology developed by Shao et al. [46] for the eastern Qaidam Basin. In doing so, we will be able to help archaeologists and historians to identify the potential candidates of the tomb owner to those who died close to the date of tomb construction.

## Materials and Methods

### 2.1 Sampling, preparation and measuring of tree-ring widths

The Reshui-1 Tomb (36.18°N, 98.3°E; 3440m a.s.l.) is situated on the north bank of the Chahanwusu River in Dulan County and is on public land (Fig 1). The tomb itself covers an area of approx. 10,240 m^2^, with a height of 27.8 m [49]. It was robbed during the period of the Republic of China (AD 1911–1949) [49]. The tomb and its affiliated relics were excavated by the Qinghai Provincial Institute of Cultural Relics and Archaeology during the period of 1982–1985. Unearthed artifacts included animal bones (horses, dogs, and cattle), a large number of silk pieces, and various gold and silver wares [4]. The tomb’s roof was built with small beams (approx. 5–15 cm in diameter) of Qilian junipers covered by loess, sand and gravel, and rocks. During a field excursion in 2013, we discovered that the north-western corner of the roof partially collapsed (Fig 1), and several Qilian juniper beams fell down to the northern base of the tomb.

**Fig 2:**
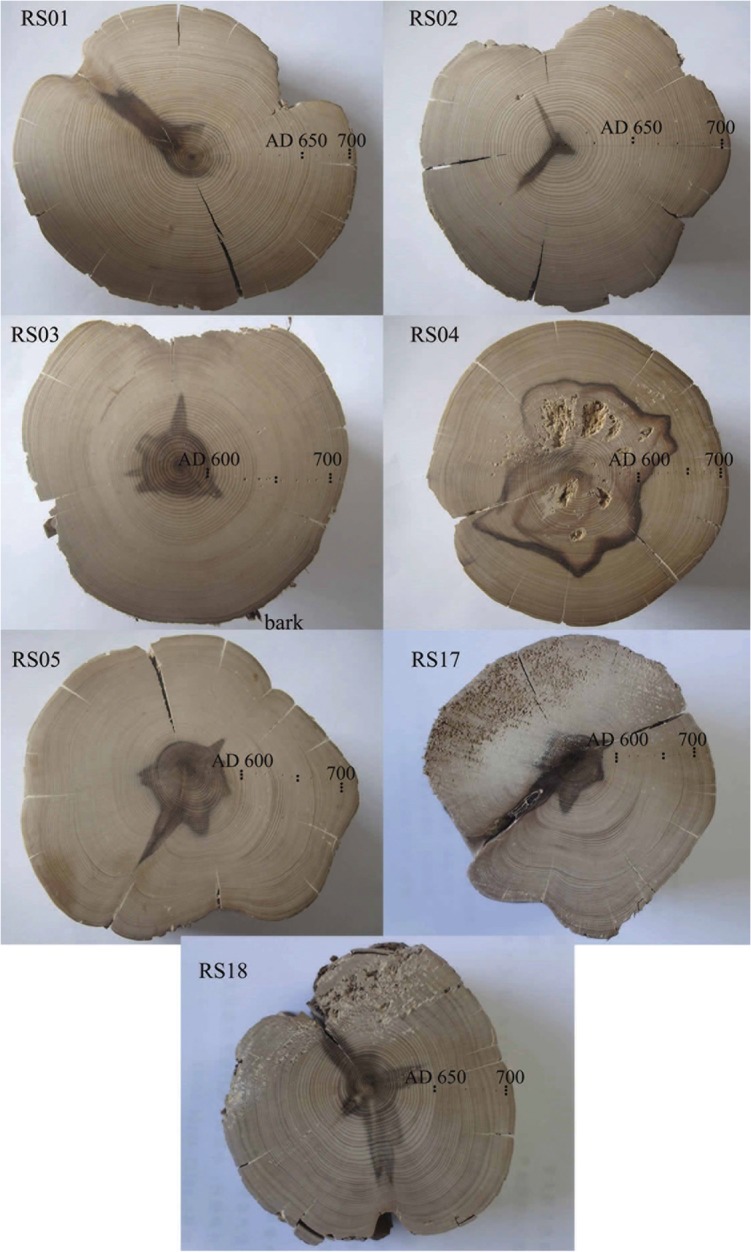
Seven Qilian juniper discs sampled from the fallen roofing beams of the Reshui-1 Tomb.

We collected seven beams fallen from the roof of the Reshui-1 Tomb and sawed one disc from each (numbered RS01-05, 17 and 18) *in situ* (Fig 2). The first five discs were collected in June 2013 and the rest two were collected in June 2014. We also took eleven increment cores (numbered RS06-16) from the exposed wood on the north-eastern side of the roof in June 2014. All specimens sampled were kept in the Tree-Ring Lab of the Institute of Geographic Sciences and Natural Resources Research, Chinese Academy of Sciences in Beijing, China. We obtained permission for fieldwork from the Qinghai Provincial Institute of Cultural Relics and Archaeology, which is in charge of all activities of archaeological research in Qinghai Province. One of the authors, Xinguo Xu, as the former director of the Qinghai Provincial Institute of Cultural Relics and Archaeology was in charge of the excavation of the Reshui-1 Tomb in the early 1980s [1]. Moreover, great caution was exercised in the field sampling process to avoid any damage to the tomb.

**Fig 3:**
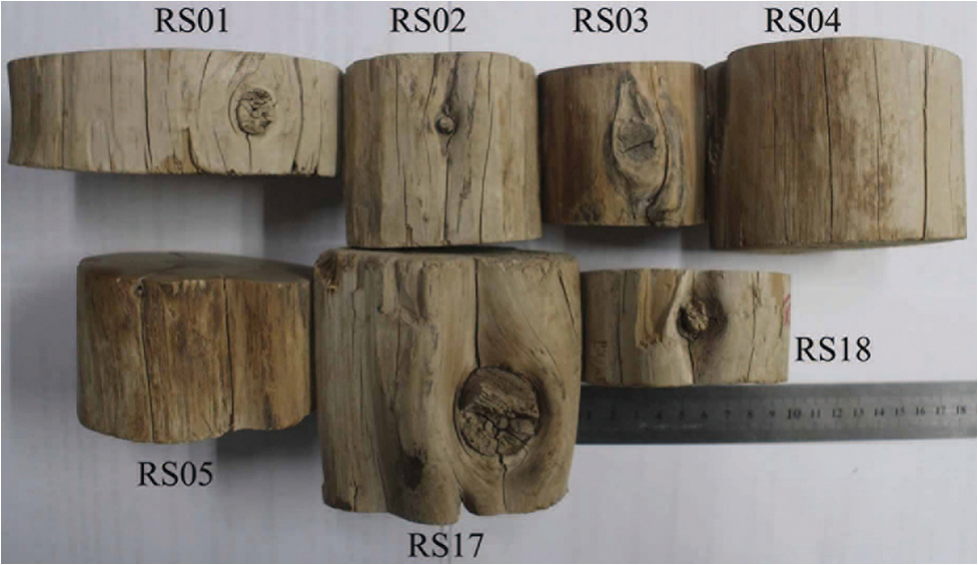
Appearances of the Qilian juniper discs sampled.

**Fig 4:**
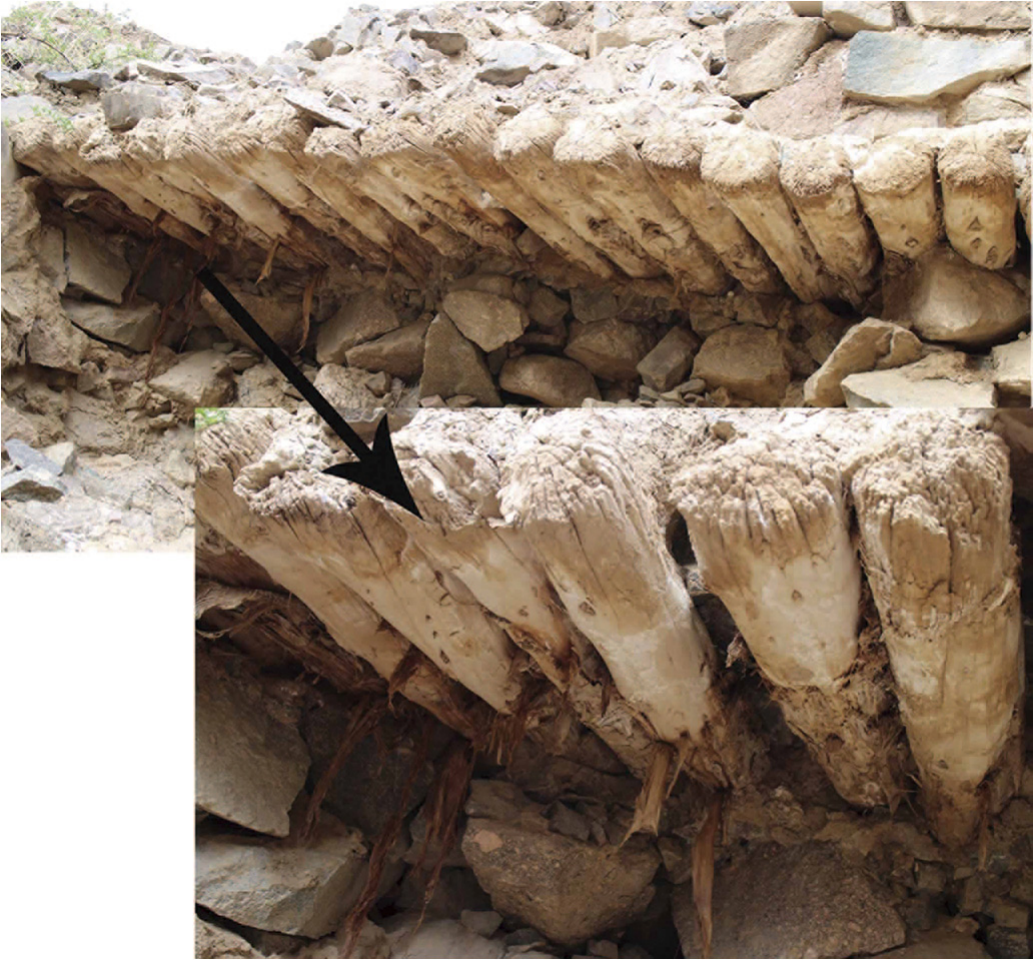
Roofing beams with barks sampled using increment borers.

The seven discs were 6.5–12 cm in diameters (Table 1) and had no barks, but based on their appearances they were not processed before they were used to build the tomb (Fig 3). The barks were probably shed when the beams were exposed in the air for a long time. The eleven cores that we sampled with increment borer still had barks attached on the outside (Fig 4). The specimens were first sanded with increasingly fine grades of sand paper, to at least 600-grit so that all cellular details of the annual rings can be seen clearly under microscopes. The tree-ring widths were measured to the nearest 0.01 mm using a Lintab ring-width measurement system. Each series was treated as a floating series with the innermost ring of each series temporarily assigned as AD 1 prior to cross-dating.

**Table 1:** Statistics of each sampled specimen.

No.	Specimen Type	No. of rings	Diameter of discs or length from pith to bark of increment cores (cm)	Max^*^ (mm)	Min^*^ (mm)	Mean^*^ (mm)	Sd^*^	MS^*^
RS1	Tree disc	111	12	1.39	0.03	0.53	0.35	0.45
RS2	Tree disc	90	7	1.23	0.03	0.42	0.27	0.42
RS3	Tree disc	125	6.5	1.17	0.02	0.29	0.19	0.46
RS4	Tree disc	148	9.5	0.9	0.02	0.33	0.21	0.56
RS5	Tree disc	152	9.8	0.87	0.03	0.30	0.17	0.37
RS6	Increment core	73	5.9	1.29	0.06	0.45	0.30	0.46
RS8	Increment core	79	7.5	1.14	0.12	0.60	0.23	0.35
RS9	Increment core	89	6.6	1.66	0.04	0.62	0.39	0.36
RS11	Increment core	125	6.5	0.85	0.11	0.42	0.16	0.17
RS12	Increment core	71	6	1.24	0.07	0.51	0.28	0.38
RS13	Increment core	124	6	1.09	0.04	0.42	0.23	0.19
RS14	Increment core	69	6.1	0.93	0.36	0.63	0.11	0.18
RS15	Increment core	121	6.3	1.09	0.23	0.44	0.14	0.20
RS16	Increment core	72	7.5	1.86	0.16	0.92	0.36	0.41
RS17	Tree disc	150	9.4	0.67	0.07	0.27	0.12	0.31
RS18	Tree disc	93	8.6	0.99	0.04	0.42	0.22	0.36

*Max: maximum width of each series; Min: minimum width of each series; Mean: average width of each series; Sd: standard deviation; MS: mean sensitivity.

### 2.2 Cross-dating

Cross-dating is the most important principle of dendrochronology. Its application provides a type of “experimental” quality control because it assures the proper placement in time of an annual growth layer [50]. Normally, the skeleton-plot technique is utilized primarily to match narrow rings among the samples to cross-date tree rings in relatively dry regions [50, 51], such as south-western North America and North-east Tibetan Plateau. Then, the COFECHA program is used to assess the quality of cross-dating and measurement accuracy of tree-ring series [52]. The COFECHA program was developed based on the concept that if all the tree ring data are perfectly dated and accurately measured, the correlation coefficients among them should be the highest comparing with series with incorrect dating or measurements [52]. Since the temporal trends contained in the data could exert certain influences on the correlation coefficients, it is preferable to first remove the low-frequency variations from all the series in practice. Then the correlations between each series and the mean series of the remaining series in the corresponding time segments are computed with a moving window shifting back and forth through time to calculate correlation coefficients. These coefficients of correlation offer the hints whether the dating and measurements are accurate. The COFECHA program also has a “cross-dated and undated” function that can be used to cross-date the undated sample series to an existing master chronology from the same region. The absolute date of a sample series is suggested when the correlation coefficient with the master chronology peaks at a given year when a moving window shifts back and forth. Good cross-dating quality is indicated by strong positive correlation coefficients that are highly significant statistically, among the sample series and between the individual sample series and the master chronology.

In this study, we used the cross-dated and undated function of COFECHA after the ring widths of the collected specimens were measured. The master dating chronology we used here was constructed previously using 1438 series from 713 trees, including samples from 22 archaeological sites, 24 living tree sites and 5 standing snags sites in the eastern Qaidam Basin [46] (as Qaidam Chronology (QC) hereafter). In order to best match the undated sample series to the existing dating chronology, we first constructed a master chronology using only specimens from the archaeological sites, known as the Qaidam Archaeological Chronology (QAC). QAC was precisely dated and well-replicated for the period before AD 800 [46], and should serve well as the master chronology for the tomb samples. Then the likely calendar dates of the sample series were assigned according to the COFECHA results. Finally, we dated each ring visually and added missing rings, if any, according to the narrow-wide ring-width patterns of the QAC. When we ran COFECHA, the program default was used, which means that all correlations were calculated after removing long-term trends in the sample series using a 32-year cubic spline function. The segment length is 50 years lagged successively by 25 years (e.g., from year 1 to year 50, and then from year 26 to year 75 until the end of the sample series) and all the series were log-transformed to enhance the influence of narrow rings. We also calculated correlations between the sample series and two more reference chronologies from Dulan [36, 40] as a separate evaluation of the cross-dating quality.

## Results

Two of the eleven increment cores (RS7 and RS10) were fragmented so badly that we could not date them. Therefore, they were not included in the following analysis. The number of rings that each specimen contains was between 69 and 152 (Table 1). The maximum width of all rings was 1.89 mm, the minimum was 0.02 mm and the average width was 0.47 mm (Table 1). The average mean sensitivity was 0.352 (Table 1), showing a similar level of year-to-year variation to that of QC (0.37) [46].

**Fig 5:**
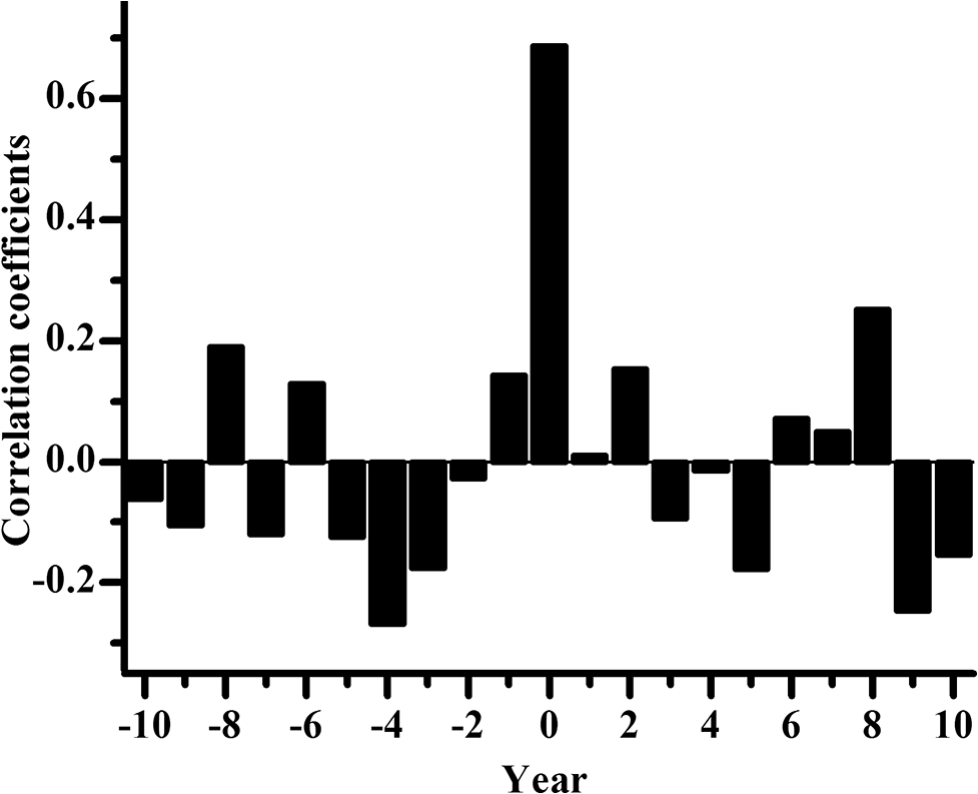
Correlation coefficients between the first 50-year segment of sample RS01 and QAC as the moving window shifted from -10 to +10 years of a potential target date with a one-year lag. Please refer to the text for details.

As described earlier, the essence of cross-dating for this study is the comparison between each individual sample series and the master dating chronology, Qaidam Archaeological Chronology (QAC). Fig 5 shows the changes in correlation coefficients between the first 50-year segment of sample RS01 and QAC (AD 605–654) as the moving window shifted from -10 to +10 years of a potential target date (year 0) with a one-year lag. Here, the correlations were calculated between RS01 and QAC successively for the 50-year window of the QAC dates from AD 595–644 (year -10) to AD 615–664 (year +10) It can be seen that as the sample series’ date matched with the master chronology at the target year (year 0 corresponding to QAC dates of AD 605–654), the correlation reached its peak value. Table 2 contains examples of COFECHA output of cross-dating for sample series RS01, RS03, and RS13. For RS01, AD 604 was the suggested calendar year prior to the first year of the sample series (Table 2). In other words, adding 604 to the years of the floating series RS01 would convert them to the calendar years, so that the first year of sample RS01 was AD 605. Ten out of the 16 sample series can be dated very easily in similar ways (RS01, 02, 05, 08, 09, 11, 14–17). For RS03, COFECHA indicated that 590 years should be added to the floating series for segments 3 and 4, so that the first calendar year of RS03 was AD 591, while adding 590 was suggested as choice #6 for the second segment (Table 2). For the first segment, however, COFECHA suggested adding 589 years to the sample years or the first sample year was assigned as AD 590 instead of 591. This was because a missing year should be added in AD 644, which would make the entire series match to the master chronology. A total of 5 samples series contained missing rings (RS03, 04, 06, 12, and 18). The sample series RS13 posed the greatest challenge in cross-dating for us. Most of the COFECHA’s top suggestions (Table 2) were far away from the timeframe indicated by previous studies (7^th^– 8^th^ century) [4, 7, 8, 26]. According to these previous studies, we picked AD 591 as the calendar year prior to the first year of the sample series and the following visual dating process proved that it was the correct year, even though it appeared only twice in the top choices provided by COFECHA (Table 2).

**Table 2:** COFECHA output showing the top six best dating adjustments (“Add”) based on the six highest correlation coefficients (“Corr #”) for RS01, 03, 13 against QAC in 50-year long segments (25-year lags). The years underlined are the final choices.

Series	Counted Segment		Add	Corr #1	Add	Corr #2	Add	Corr #3	Add	Corr #4	Add	Corr #5	Add	Corr #6
RS01	1	50	604	0.69	-127	0.55	-352	0.54	-616	0.51	-840	0.49	193	0.48
RS01	26	75	604	0.66	-616	0.47	-396	0.47	-992	0.47	504	0.44	381	0.44
RS01	51	100	604	0.72	-53	0.57	-119	0.52	-1320	0.50	-854	0.47	-802	0.47
RS01	62	111												
	Add No R_av													
	+604 3 0.69													
Series	Counted Segment		Add	Corr #1	Add	Corr #2	Add	Corr #3	Add	Corr #4	Add	Corr #5	Add	Corr #6
RS03	1	50	589	0.52	-1569	0.46	549	0.45	310	0.44	-1232	0.44	-580	0.42
RS03	26	75	219	0.53	617	0.5	-487	0.48	582	0.46	-632	0.46	590	0.45
RS03	51	100	590	0.76	-67	0.50	-1443	0.49	-1470	0.46	-632	0.45	413	0.43
RS03	76	125	590	0.67	-67	0.53	-964	0.47	-816	0.47	-350	0.46	-1052	0.46
	Add No R_av			Add No R_av										
	-67 3 0.48			+590 3 0.63										
Series	Counted Segment		Add	Corr #1	Add	Corr #2	Add	Corr #3	Add	Corr #4	Add	Corr #5	Add	Corr #6
RS13	1	50	-267	0.58	59	0.48	591	0.47	-35	0.43	-152	0.43	288	0.42
RS13	26	75	354	0.52	-423	0.47	-421	0.44	-545	0.43	-1567	0.42	591	0.41
RS13	51	100	-358	0.45	322	0.45	-1123	0.43	-237	0.42	354	0.41	491	0.40
RS13	75	124	-494	0.56	-87	0.51	393	0.49	322	0.47	-1306	0.43	-696	0.43
	No pattern found													

Table 3 summarizes the results of cross-dating for all sample series, including the suggested years prior to the first sample years and the corresponding correlation coefficients for the floating series. It can be seen that COFECHA provided good suggestions to the ages for most of the floating sample series. The absolute ages of the sixteen series (Table 3) range from AD 564 to AD 715. There are eight missing rings in the sixteen specimens and AD 681 was seen in three sample series as a year of missing rings (RS04, RS06 and RS18), while RS18 had the most missing rings (AD 681, 691, 700, 711) (Table 3). The overall percentage of missing ring is only 0.471% for our samples. Previous studies have found relatively high percentages of missing rings in moisture sensitive ring-width series in this region [36–37, 40]. The low frequency of occurrence of missing rings in our samples is probably mostly due to the young ages of these trees [50].

**Table 3:** Suggested years from COFECHA output for the sixteen sample series and record lengths, time spans, and the years of missing rings of each series.

Sample	Total No. segment	First suggestion			Second suggestion			Recording length	Time span	Year of missing ring
		Add year	No. segment	Corr.	Add year	No. segment	Corr.			
RS01	3	604	3	0.69				111	605–715	none
RS02	3	625	3	0.63				90	626–715	none
RS03	4	-67	3	0.48	590	3	0.63	126	590–715	644
RS04	5	566	4	0.60				149	567–715	681
RS05	5	563	5	0.59				152	564–715	none
RS06	2	633	1	0.66				74	634–707	681
RS08	2	628	2	0.69				79	629–707	none
RS09	3	-1184	3	0.43	626	3	0.73	89	627–715	none
RS11	4	590	3	0.67				125	591–715	none
RS12	2	592	2	0.54				72	593–664	654
RS13^*^	4	591	2	0.44				124	592–715	none
RS14	2	643	2	0.56				69	644–712	none
RS15	4	594	4	0.54				121	595–715	none
RS16	2	607	2	0.69				72	608–679	none
RS17	5	565	5	0.64				150	566–715	none
RS18	3	618	2	0.64				97	619–715	681, 693, 700, 711

*: To date the sample RS13 we chose AD 591 as a suggestion year according to the COFECHA output, although no clear pattern was found in Table 2. Please see the text for details.

Table 4 shows the final results from the COFECHA correlation analysis between AD 550 and AD 724 for the dated sample series. It contains the correlation coefficients for each 50-year segments with 25-year lags for each sample series and the mean correlation coefficients with the COFECHA master series, which were used to evaluate the quality of cross-dating. All sixteen series had statistically significant correlations for all 50-year segments tested and the correlations ranged from 0.41 to 0.88. Inter-series correlations (the correlation of one series against a composite created from the other fifteen series) ranged from 0.498 to 0.830. The average inter-series correlation was 0.696 over the full periods (Table 4), suggesting strong consistency among the 16 dated sample series.

**Table 4:** Results from the COFECHA correlation analysis conducted for tree-ring measurements of the sixteen sample series from the Reshui-1 Tomb.

Sample	Time span	50-year segment tested (lagged by 25 years)						Correlation with master
		550–599	575–624	600–649	625–674	655–699	675–724	
RS01	605–715			0.80	0.81	0.84	0.88	0.816
RS02	626–715				0.54	0.72	0.80	0.669
RS03	590–715		0.48	0.58	0.69	0.79	0.77	0.697
RS04	567–715	0.70	0.77	0.84	0.80	0.73	0.70	0.747
RS05	564–715	0.79	0.75	0.68	0.65	0.59	0.65	0.697
RS06	634–707				0.79	0.75	0.74	0.830
RS08	629–707				0.80	0.83	0.80	0.820
RS09	627–715				0.76	0.80	0.86	0.827
RS11	591–715		0.42	0.50	0.64	0.64	0.66	0.561
RS12	593–664		0.75	0.75	0.74			0.739
RS13	592–715		0.56	0.51	0.43	0.41	0.50	0.498
RS14	644–712				0.60	0.62	0.73	0.645
RS15	595–715		0.71	0.64	0.44	0.62	0.67	0.645
RS16	608–679			0.79	0.79	0.79		**0.752**
RS17	566–715	0.70	0.70	0.76	0.61	0.56	0.60	0.649
RS18	619–715			0.66	0.69	0.72	0.50	0.707
Average correlation		0.73	0.64	0.68	0.67	0.69	0.70	0.696

**Fig 6:**
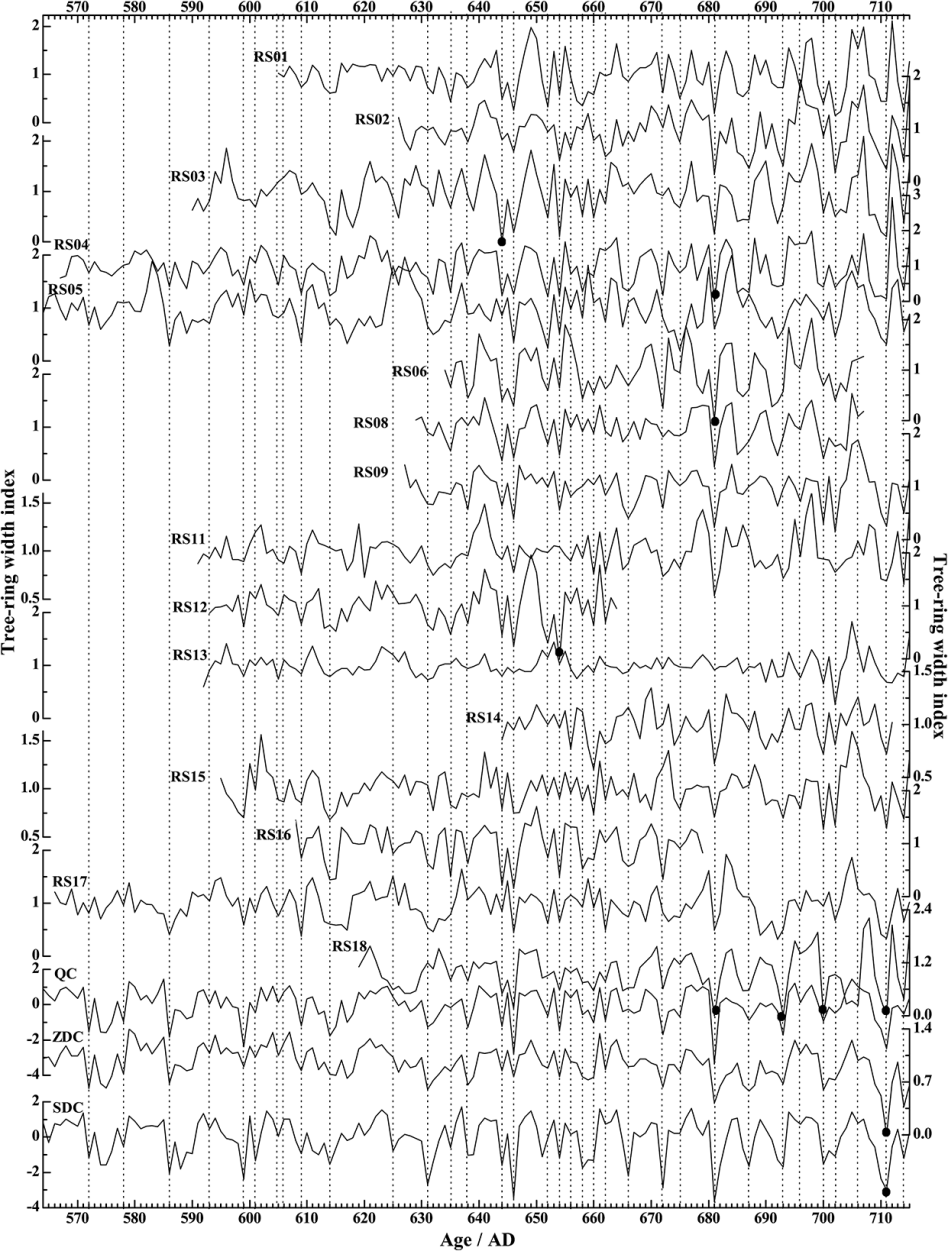
Time-series plots of the sixteen dated sample series and three reference chronologies; the vertical dotted lines mark years with narrow rings. RS01-06, 08, 09, 11–18: The 16 dated series from archaeological wood sampled; QC: The Qaidam Chronology constructed by Shao et al. [46]; ZDC: The Dulan chronology constructed by Zhang et al. [36]; SDC: The Dulan chronology constructed by Sheppard et al. [40]. The black dots show the locations of the missing rings.

**Fig 7:**
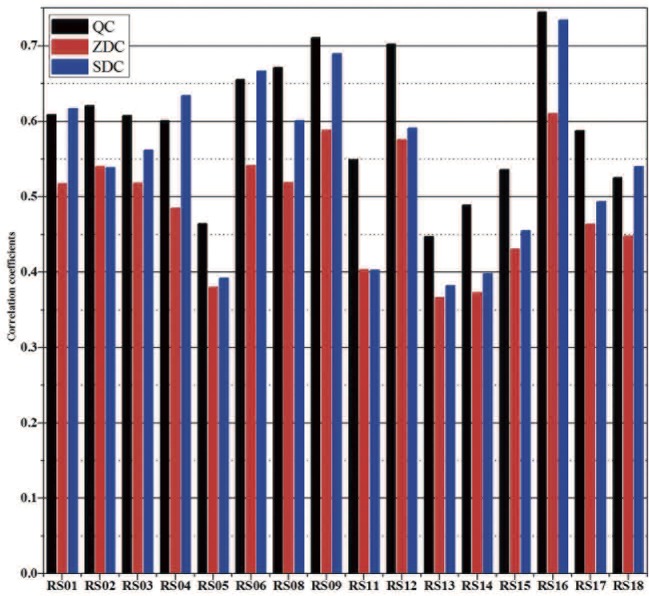
Correlation coefficients between the 16 dated sample series and the three reference chronologies (QC, ZDC, and SDC).

The quality of cross-dating can also be illustrated by Fig 6 that contains the time-series plots of the 16 dated sample series. It can be seen that the variation patterns of the 16 ring-width series exhibit an excellent match among themselves, especially for the years with narrow rings. Also plotted in Fig 6 is the Qaidam Chronology (QC) together with two additional chronologies adjusted based on QC [46] from Dulan, one constructed by Zhang et al. [36] (as ZDC) and the other by Sheppard et al. [40] (as SDC). Again, the match between the 16 series to these reference chronologies was also very good (Fig 6). Fig 7 shows the correlation coefficients between the sample series and the three reference chronologies. Sample series RS16 has the highest correlations with the three chronologies (r = 0.745 to QC, 0.611 to ZDC, and 0.734 to SDC), while the lowest correlations were found for sample series RS13 (r = 0.447 to QC, 0.366 to ZDC and 0.382 to SDC). The mean correlation coefficients to the reference chronologies are 0.595 to QC, 0.485 to ZDC, and 0.544 to SDC, respectively.

As stated above, the latest year for most of the 16 dated series was AD 715 (Table 3). In fact, seven discs and four out the nine increment cores all ended in this year (Fig 6). For the remaining five increment cores (RS06, 08, 12, 14, and 16), the outermost rings were decayed due to weathering and the latest years that had been dated are AD 707, 707, 664, 712, and 679, respectively. To make sure that the missing of the outermost rings in these samples was not due to wood processing, we carefully examined the morphology of the discs and increment core samples. Fig 3 shows the conditions of the 7 wood discs and it can be seen that there are no signs of wood processing, which means that the outermost rings of these discs should represent the years when the trees were cut. In the meantime, we observed in the field that barks were still attached to some of the beams sampled by increment borers (Fig 4), so that the outermost rings of these samples should also represent the years when the trees were felled. Since the ages of the outermost rings from eleven out of the sixteen trees are AD 715 (7 discs and 4 increment cores), it can be concluded that all these trees sampled were not processed before they were used to build the tomb, which is consistent with the earlier observations of wood used in other ancient tombs in the area [11]. In addition, the latewood of the outermost rings of the RS11 and 13 was visible under the microscopes. Therefore, these trees must have been cut down in late AD 715 or before the growing season started in AD 716.

## Discussions

The dates of ancient events and ages of artifacts have important meanings in archaeology. For example, determining the ages and construction histories of monuments is important to relate religious symbolism, scientific knowledge, and cultural continuity to groups within a region [53]. It is well known that tree-ring data have the advantages of accurate dating, annual resolution, wide availability, and good replication [50]. Prehistoric sites and ancient tombs that contained wood specimens can be dated using regional reference tree ring chronologies, but it can be difficult at times due to the lack of well-preserved rings and collaborations with field archaeologists are often necessary to achieve accurate dating results [18, 22]. The dry climate in the eastern Qaidam Basin provides the optimal condition for preservation of wood [26, 46, 54]. As shown in Fig 4, the barks are still attached to most of the beams we sampled using increment borers, showing low degrees of decay and weathering after more than 1000 years of tree harvesting.

COFECHA was very effective in providing fairly accurate suggested ages for most sample series in our study (Table 3). When comparing with other studies that employed COFECHA, our cross-dating results apparently have a similar level of quality. For example, the correlations of the sample series segments with the COFECHA master series from our study ranged from 0.41 to 0.88 (Table 4), while those in Grissino-Mayer et al. [12] are from 0.19 to 0.76. For the mean correlation with the COFECHA master series, our result is from 0.498 to 0.830, while those in Therrell and Stahle [55] is from 0.190 to 0.732.

In the light of the local tradition, the beams should have been used to build the tomb after the trees were cut down for about ten days [7]. Therefore, we conclude that the tomb was built using fresh wood soon after the trees were harvested. As to the 5 increment cores dated prior to AD 715 even with barks still remaining on the beams, the wood was weathered over time and very fragile, so the rings immediately beneath the bark fell off at the time of sampling by the increment borer. Therefore, it is reasonable to conclude that the Reshui-1 Tomb was completed in late AD 715 or early AD 716, since the samples were taken from the roofing beams whose installation should be the last step of tomb construction. That is to say, the Reshui-1 Tomb was finished in the Tubo Kingdom Period. This date was consistent with those estimated ages (ranging from the second half of the 7^th^ century to the first half of the 8^th^ century) based on the unearthed artifacts from the Reshui-1 Tomb [4, 6], which lead to the speculations that the tomb owner was of Tubo ethnicity.

Wang et al. [8] used dendrochronology to date 7 tombs of the Reshui Tomb Cluster. They determined that one tomb (numbered 00DRXM10) was built in AD 611 belonging to the Tuyuhun Kingdom Period, while the other six tombs were built in the Tubo age after AD 663. The Dulan area was the territory of the Tuyuhun Kingdom during AD 329–663 and Dulan was the capital of the Tuyuhun Vassal State of the Tubo Kingdom after AD 663 [5]. Therefore, the Reshui Tomb Cluster was probably the relics of the people of Tuyuhun ethnicity [1, 4, 6]. Others argued that the Reshui Tomb Cluster was started by the Tuyuhun people but later was continued by the Tubo people after AD 663, while the ancient Tombs in Delingha during the same period exclusively belonged to the Tuyuhun people [56].

Our analysis results suggested that the trees were harvested in late AD 715 or early AD 716 before the growing season started. According to the ancient Tibetan inscribed wooden slips and coins of the Tang Dynasty (Kaiyuan Tongbao or Currency, first minted in AD 621) unearthed from the Reshui-1 Tomb excavation, it was determined that the tomb was built during AD 713–741 [6], which would imply the tomb owner as a member of the Tubo Kingdom. On the other hand, Tong [4] dated the Reshui-1 Tomb to the time from the end of the 7^th^ century to the beginning of the 8^th^ century based on silk pieces, color-painted wooden planks, and gold and silver wares unearthed from the tomb, and speculated that the owner of the tomb was a Tuyuhun King (Khan) established by the Tubo Kingdom after AD 663. However, Tong [4] also pointed out that this Tuyuhun Khan Bendayanchisong (dBon da rgyal khri zung) died in AD 694. So it is unlikely that his tomb was finished 21 years after his death, as the deceased should be buried soon after the death according to the local funeral tradition during the Tubo and Tuyuhun age [57]. Even if we consider the uncertainties in the exact year of his death (AD 689–706) [4], it was still at least approximately 10 years before the completion of the Reshui-1 Tomb. The exact date of the tomb construction plus the knowledge of the tomb structure and unearthed artifacts from the Reshui-1 Tomb indicated that the tomb owner was someone of high importance, either as a Tuyuhun under Tubo’s ruling or a member of the Tubo Kingdom. Hence, we still cannot determine the exact date of tomb usage or the tomb owner’s time of death at this point. If we want to know more about the exact ethnicity and identify of the Reshui-1 Tomb, we need to comprehend the social and political characteristics of the Tuyuhun-Tubo age and have a more complete survey of the ages of the ancient tombs in this area with the help from archaeologists. We also need a thorough investigation on historical documents from both Tibetan and Han Chinese sources, which may provide the times of death of important people in this region around AD 715 or 716.

## Conclusions

In this study, we sampled 18 roofing beams from the Reshui-1 Tomb, the largest tomb of the Tuyuhun-Tubo ages in Qinghai Province, and 16 of them could be accurately dated. Based on cross-dating to an existing master dating chronology, the Qaidam Chronology, we dated the roofing beams of the Reshui-1 Tomb as being harvested in late AD 715 or before the growing season started in AD 716, and the Tomb itself was probably finished soon after and belonged to the Tubo age. This date was supported by high-quality cross-dating among the sample series themselves and between these series and three reference chronologies from the study area. Our results corroborated with previous archaeological studies and settled the question whether the Tomb belonged to the Tuyuhun or Tubo age. As to the tomb owner’s identify, the ethnicity of the tomb owner still could not be determined at this moment. The same is true for the exact date of tomb usage or the time of death of the tomb owner. Additional archaeological analysis and research of historical documents need to be performed to further our understanding of the history of this unique cultural relic.
